# Modeling a Production Function to Evaluate the Effect of Medical Staffing on Antimicrobial Stewardship Performance in China, 2009–2016: Static and Dynamic Panel Data Analyses

**DOI:** 10.3389/fphar.2018.00775

**Published:** 2018-07-16

**Authors:** Junjie Liu, Chun Yin, Chenxi Liu, Yuqing Tang, Xinping Zhang

**Affiliations:** ^1^School of Medicine and Health Management, Tongji Medical College, Huazhong University of Science and Technology, Wuhan, Hubei, China; ^2^The Center for Modern Chinese City Studies & School of Urban and Regional Science, East China Normal University, Shanghai, China

**Keywords:** antimicrobial stewardship, medical staffing, production function, panel data econometrics, fixed-effect model, dynamic model

## Abstract

**Background:** Antimicrobial resistance (AMR) is an international problem. Emergence and spread of AMR are strongly associated with overuse or inappropriate use of antimicrobials. Antimicrobial stewardship ensures the appropriate use of antimicrobials, and is an effective approach to control AMR. This study aims to understand the relationship between medical staffing and antimicrobial stewardship performance in China.

**Methods:** A provincial-level panel dataset from 2009 to 2016 is used. A macro production function is used to quantify the relationship. The output, antimicrobial stewardship performance, is measured by changes in methicillin resistance rates of *Staphylococcus. aureus* (*S. aureus*) and coagulase-negative staphylococci (CoNS). The labor input is measured by the numbers of infectious diseases physicians, pharmacists, clinical microbiologists, and nurses in hospitals per 100,000 populations, whereas the capital input is represented by the number of hospital beds per 100,000 populations. The technology is captured by the time index. Both static and dynamic panel data approaches are employed.

**Results:** The increasing number of clinical microbiologists is a significant predictor of lower resistance of CoNS according to dynamic models (Coef. = −0.191, −0.351; *p* = 0.070, 0.004, respectively). However, a larger number of nurses is significantly associated with higher resistance of *S. aureus* (Coef. = 0.648; *p* = 0.044). In addition, the numbers of the other two groups of medical professionals exhibit no significant associations with stewardship performance.

**Conclusions:** The study demonstrates the crucial role of clinical microbiologists in antimicrobial stewardship. The predicted increased risk of resistance with the higher number of nurses may be attributable to their lack of related knowledge and their unrecognized functions in antimicrobial stewardship.

## Introduction

Antimicrobial resistance (AMR) is an international public health crisis. Once pathogenic microbes develop resistance to antimicrobials, the options for treating infections that they cause are reduced (World Health Organization, 2015). Thus, AMR accounts for the loss of antimicrobial effectiveness and threats the capability of modern medicine to cure infectious diseases (Laxminarayan et al., 2016). Dramatic increases in AMR have been witnessed in recent years, costing numerous lives around the globe (Dellit et al., 2007). More than 700,000 people die annually from infectious diseases which are attributable to resistant pathogens (O'Neill, 2016). This number will predictably balloon to 10 million in 2050 if no action is taken (O'Neill, 2016). The emergence and spread of AMR are strongly associated with overuse or inappropriate use of antimicrobials (Goossens, 2009; Van Boeckel et al., 2014; Li et al., 2017).

The development of new antimicrobials has been vastly outpaced by the prevalence of AMR (Brooks and Brooks, 2014). This condition is attributed to two main reasons. Firstly, antimicrobial development becomes technically harder today than it was before (Owens, 2008). More importantly, antimicrobial development suffers from decades of under-investment (O'Neill, 2016). In total, 38 billion US dollars venture capital was invested into pharmaceutical research and development in 2003–2013; however, less than 5% was allotted for antimicrobial development (O'Neill, 2016; Renwick et al., 2016).

Hence, as AMR increases, and development of new antimicrobials declines, it is imperative to make the best use of currently available antimicrobials to extend their useful lifetime (MacDougall and Polk, 2005; Owens, 2008). This condition highlights the importance of antimicrobial stewardship. This practice ensures the appropriate use of antimicrobials (e.g., the optimal selection, dose, and duration of antimicrobial therapy) to optimize clinical outcomes, ensure cost-effectiveness, and minimize unintended consequences of antimicrobial use, especially the emergence of AMR (MacDougall and Polk, 2005; Owens, 2008; Patel et al., 2008; Dik et al., 2016; Dyar et al., 2017). Antimicrobial stewardship has been widely conducted in different countries.

Medical staffing is essential to antimicrobial stewardship. Considering that AMR is a multifaceted problem, and comprehensive and multi-modal strategies of antimicrobial stewardship should be established, a collaborative stewardship team including medical professionals with multidisciplinary backgrounds is recommended (Marr et al., 1988; Paskovaty et al., 2005). Usually, infectious diseases physicians, pharmacists, and clinical microbiologists serve as the core members of the stewardship team (Paskovaty et al., 2005; Kim et al., 2015; Pulcini et al., 2017). Other professionals such as nurses also play essential roles (Olans et al., 2016; Monsees et al., 2017). The team implements antimicrobial stewardship and optimizes the clinical use of antimicrobials and infection management (Pulcini et al., 2017).

The relationship between medical staffing and health care-related outcomes is a significant concern in the field of health service research and health economics. Prior empirical studies are conducted from a micro or macro perspective. From the micro perspective, department-level or hospital-level data are consistently used. For instance, Liang et al. (2015) estimated the relationship between nurse staffing, which was measured by the patient-to-nurse ratio, and patient mortality in 108 hospital nursing units in Taiwan. They observed that nursing units with a patient-to-nurse ratio higher than 8 exhibited significantly higher patient mortality. Mark et al. (2004) examined the relationship between the number of full-time equivalent registered nurses (RNs) per 1,000 inpatient days and quality of care measured by patient mortality in 422 hospitals. Interestingly, their results showed the diminishing marginal effect of increasing nurse staffing on reducing patient mortality.

From the macro perspective, state-level or country-level data are used. Huang et al. (2016) estimated the relationship between medical staffing, which was measured by the sum per thousands of RNs and physicians, and life expectancy in Taiwan by using aggregated time series data. Suraratdecha and Okunade (2006) investigated the relationship between medical staffing and health system performance in five regions in Thailand. The medical staffing was measured by the numbers of physicians, nurses, and pharmacists, whereas the performance was measured by the number of live births per 1,000 population. They reported that only nurses enjoyed the positive effects on the performance, whereas higher numbers of physicians and pharmacists were associated with worse performance. Okunade (2001) focused on pharmacy sector and estimated the relationship between the number of pharmacy personnel and pharmacy costs with aggregated time series data of US hospital pharmacies. He noted that increasing involvements of pharmacists contributed to decreased costs in several specific pharmaceutical care services.

Focusing on the relationship between medical staffing and antimicrobial stewardship performance, most studies are conducted from micro perspectives (Dranitsaris et al., 2001; Schouten et al., 2005; Apisarnthanarak et al., 2015). Relatively few studies are from macro perspectives. As macro studies go beyond details of the behavior of individual units, they facilitate increased understanding of underlying connections among systems (Dornbusch et al., 2002). Therefore, macro studies bear empirical significance in antimicrobial stewardship performance.

Moreover, few studies pay attention to the relationship between medical staffing and antimicrobial stewardship performance in developing countries such as China, in which antimicrobial overuse and AMR are common (Mao et al., 2015; Ma et al., 2016). The Chinese government highlights the antimicrobial stewardship especially the establishment of an antimicrobial stewardship team. Three core groups of medical professionals, namely, infectious diseases physicians, pharmacists and clinical microbiologists, should lead antimicrobial stewardship in hospitals (National Health and Family Planning Commission, 2015a). Their practice scopes are defined as follows: infectious diseases physicians guide their colleagues in prescribing antimicrobials within a hospital; pharmacists advise physicians in prescribing and guide patients to take antimicrobials with their pharmaceutical knowledge; clinical microbiologists conduct microbial cultivation, isolation, antimicrobial susceptibility testing and reporting to provide rationale for antimicrobial selection (National Health and Family Planning Commission, 2015a).

Although the Chinese government has recorded remarkable achievements in antimicrobial stewardship, it also faces several problems, specifically regarding medical staffing (National Health and Family Planning Commission, 2016a). Firstly, the government realizes the inadequacy of infectious diseases physicians in China. As most infectious diseases physicians in China focus mainly on notifiable communicable diseases such as viral hepatitis and tuberculosis, and receive no sufficient training in clinical microbiology and infection control, they lack abilities to diagnose and treat bacterial and fungal infections, preventing them from guiding their colleagues in other clinical departments in antimicrobial use (National Health and Family Planning Commission, 2016a; Zhang et al., 2018). Secondly, the number of pharmacists who are capable of providing physicians suggestions on prescription and promoting the prudent use of antimicrobials is inadequate (Penm et al., 2014; National Health and Family Planning Commission, 2016a). These pharmacists are also unevenly distributed across the health sector with more than half of them concentrated in the more developed Eastern regions of China (Fang, 2016; National Health and Family Planning Commission, 2016a).

To address the above-mentioned gaps, the objective of this study is to estimate the relationship between medical staffing and antimicrobial stewardship performance in China from a macro perspective. A provincial-level panel dataset from 2009 to 2016 is used. The production function is employed to model the relationship. The study is expected to provide policymakers, especially those in developing countries, macro evidence on the allocation, utilization, and management of human resources for antimicrobial stewardship.

## Materials and methods

### Production function

The production function is a key concept in economics. This function exhibits the quantitative relationship between inputs and outputs (Dornbusch et al., 2002). The generic formula for production function is written as follows:

(1) $$\begin{array}{l} Q\left(t\right)=A\left(t\right)*F\left[K\left(t\right),L\left(t\right)\right] \end{array}$$

where *Q*(*t*) denotes the output at time *t*, and *K*(*t*) and *L*(*t*) refer to capital and labor input at time *t*, respectively. *A*(*t*) is the level of technology.

Cobb–Douglas production function is one of the most commonly used and specific forms of production function (Dornbusch et al., 2002). This function is written as follows:

(2) $$\begin{array}{l} Q\left(t\right)=A\left(t\right){K\left(t\right)}^{\alpha }{L\left(t\right)}^{\beta } \end{array}$$

Logarithm transformation of Cobb–Douglas production function into linear form yields the following:

(3) $$\begin{array}{l} \ln Q\left(t\right)=\ln A\left(t\right)+\alpha \ln K\left(t\right)+\beta \ln L\left(t\right) \end{array}$$

Coefficients α and β are the output elasticities of capital and labor inputs, respectively. Output elasticity is defined as the ratio of the percentage change in the output to the percentage change in an input, reflecting the contribution of the input to the output (Hongye, 2014).

We employ production function to estimate the relationship between medical staffing and antimicrobial stewardship performance. In this framework, the output is antimicrobial stewardship performance, and the labor input is medical staffing. Hence, coefficient β captures the effect of medical staffing on the stewardship performance.

### Data sources and variable measurement

The present study uses annual provincial panel data from 2009 to 2016. The data originate from two sources: (1) *China's Statistics Yearbooks of Health and Family Planning* 2010–2017 (Ministry of Health, 2010, 2011, 2012; National Health and Family Planning Commission, 2013, 2014, 2015b, 2016b, 2017), and (2) *CHINET Surveillance Reports of Bacterial Resistance* 2009–2016 (Wang et al., 2010, 2013; Zhu et al., 2011; Hu et al., 2012, 2014, 2015, 2016b, 2017). The former is published by the National Health and Family Planning Commission. They offer annual statistics on a range of health care-related issues, such as health care facilities, human resources for health, and epidemiology of different diseases in China. The statistics reflect the development of health care system and citizens' health status in China (National Health and Family Planning Commission, 2017). The latter is published by CHINET surveillance system, an organization which gathers AMR patterns from multiple clinical laboratories in China and focuses on AMR surveillance (Hu et al., 2016a). These reports publish annual AMR statistics, such as the distribution of common resistant strains and resistance rates of strains to different classes of antimicrobials. The statistics are obtained from the clinical laboratories of the largest hospitals in different provinces or cities, and objectively reflect AMR patterns in the corresponding places (Hu et al., 2016a).

To model the production function of antimicrobial stewardship performance, we collect data from the two data sources and set the variables. Table 1 provides the detailed information on the variable definitions and sources.

**Table 1:** Variable definitions and sources.

**Variables**	**Definitions**	**Sources**
**OUTPUT**		
*STW*	(1) Methicillin resistance rate of *S. aureus*; (2) Methicillin resistance rate of coagulase-negative staphylococci (CoNS);	CHINET Surveillance Reports of Bacterial Resistance
**LABOR INPUT**		
*INF*	The number of infectious diseases physicians in hospitals per 100,000 populations	China's Statistics Yearbooks of Health and Family Planning
*PHR*	The number of pharmacists physicians in hospitals per 100,000 populations	China's Statistics Yearbooks of Health and Family Planning
*MCB*	The number of clinical microbiologists in hospitals per 100,000 populations	China's Statistics Yearbooks of Health and Family Planning
*NRS*	The number of nurses in hospitals per 100,000 populations	China's Statistics Yearbooks of Health and Family Planning
**CAPITAL INPUT**		
*BED*	The number of hospital beds per 100,000 populations	China's Statistics Yearbooks of Health and Family Planning
**TECHNOLOGY**		
*T*	Time index (*T* = 1, 2, …, 8)	–

*S. aureus refers to Staphylococcus aureus*.

The output is measured by methicillin resistance rates of *S. aureus* and coagulase-negative staphylococci (CoNS). The rates are the proportions of methicillin resistance among *S. aureus* and CoNS isolates, respectively. Changes in the resistance rates reflect the performance of antimicrobial stewardship (Patel et al., 2008; Dik et al., 2016).

The labor input is measured by four variables: the numbers of infectious diseases physicians, pharmacists, clinical microbiologists, and nurses in hospitals per 100,000 populations. The numbers of infectious diseases physicians and clinical microbiologists are not directly given in the yearbooks. We make an approximation and calculate the numbers based on the available data in the yearbooks, namely, the total number of physicians in each province and the specialty distributions of physicians in infectious diseases department and clinical laboratory, respectively. In addition to the three core groups, we also consider nurses because they account for the largest clinical discipline, and always serve as executors of antimicrobial stewardship (Olans et al., 2016; Monsees et al., 2017). Dividing the numbers by 100,000 populations adjusts the population for their health care demand (Tanihara et al., 2011; Hara et al., 2018).

The capital input is measured by the number of hospital beds per 100,000 populations. The quantity of hospital beds is a commonly used variable of hospital capital input (Mark et al., 2004; Suraratdecha and Okunade, 2006; Hadji et al., 2014). Similar with the variables of labor input, the number of hospital beds is also divided by 100,000 populations.

The technology is proxied by a time index (Di Matteo, 2005; Chen et al., 2014; Beilfuss and Thornton, 2016; Khan et al., 2016). The time index captures developments in the treatments, procedures, and devices which may be used to prevent, diagnose, and treat health problems (Baltagi et al., 2012).

### Empirical analysis

As a starting point, we conduct a conventional static panel data analysis. Fixed-effect (FE) model is employed to estimate the production function based on Equation (3). The FE model is given as follows:

(4) $$\begin{array}{l} \ln\;{STW}_{it}=\beta_{0}+\beta_{1}\ln\;{INF}_{it}+\beta_{2}{\ln\;PHR}_{it}+\beta_{3}{\ln\;MCB}_{it}\\ +\beta_{4}\ln\;NRS_{it}+\beta_{5}\ln\;{BED}_{it}+\delta T_{t}+\eta_{i}+\epsilon_{it} \end{array}$$

where the subscript *it* denotes province *i* (*i* = 1, 2, …, 31) at time *t* (*t* = 1, 2, …, 8). η_*i*_ is the unobserved province-specific fixed effect, and ϵ_*it*_ is the random error.

Although the static approach is widely used, it has some limitations. Firstly, this approach neglects the dynamic nature of panel data, causing bias (Wawro, 2002). That is, the static approach assumes that stewardship performance is explained solely by contemporary inputs and technology. However, in fact, while contemporary inputs and technology notably affect stewardship performance, historical performances also cause effects (Mark et al., 2004; Aakvik and Holmas, 2006). To address this problem, we include lags of the dependent variable as regressors in the model. Secondly, the static approach assumes that all inputs are strictly exogenous, that is, they are uncorrelated with ϵ_*it*_ in all time periods. Nevertheless, “feedback effects” always exist and accounts for the endogeneity across time periods (Mark et al., 2004). For instance, the stewardship performance at time *t* may affect the labor input at time *t* + 1. Hence, we formulate a weaker assumption that inputs are “predetermined”: ϵ_*it*_ is uncorrelated with the past and current values of inputs, whereas it is potentially correlated with the future values of inputs (Mark et al., 2004; Wintoki et al., 2012).

Considering the above limitations of the static approach, the dynamic approach is employed. The dynamic model is given by the following:

(5) $$\begin{array}{l} \ln\;{STW}_{it}=\beta_{0}+\overset{m}{\underset{k\;=\;1}{\sum }}\gamma_{k}\ln\;STW_{i,t-k}+\beta_{1}\ln\;{INF}_{it}\\ +\beta_{2}{\ln\;PHR}_{it}+\beta_{3}\ln\;MCB_{it}+\beta_{4}\ln\;NRS_{it}\\ +\beta_{5}\ln\;BED_{it}+\delta T_{t}+\eta_{i}+\epsilon_{it} \end{array}$$

where *m* denotes the maximum lag order of the dependent variable. Previous studies suggest that two lags are sufficient to ensure dynamic completeness (Wintoki et al., 2012). Hence, we set *m* = 1 and *m* = 2 in our study.

The “feedback effects” and the inclusion of lagged independent variables cause inconsistency in standard estimators. To obtain consistent estimators, one-step system generalized method of moments (GMM-SYS) technique is employed (Arellano and Bond, 1991; Judson and Owen, 1999). The validity of GMM-SYS estimators is examined by two key tests. The first is Arellano–Bond test which examines the hypothesis that ϵ_*it*_ is not serially correlated by testing the serial correlation of differenced residuals. The residuals should be correlated in the first difference *AR*(1) but uncorrelated in the second *AR*(2) (Wintoki et al., 2012). The second test is Sargan test of over-identification. Failure of Sargan test to reject the null hypothesis indicates the validity of instruments (Khadraoui, 2012).

As better stewardship performance means lower resistance rates, all inputs and the technological progress are envisaged to show negative estimates in both static and dynamic panel data analyses.

RStudio (version 3.4.3, RStudio, Massachusetts, USA) is used to construct figure images. STATA (version 13.0, Stata Corp., College Station, Texas, USA) is used for statistical analysis.

## Results

### Summary statistics

**Figure 1:**
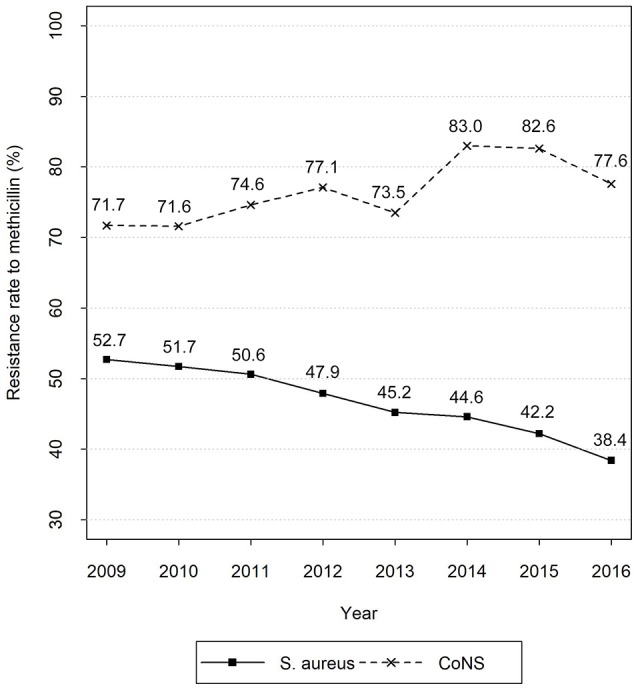
Time series of average methicillin resistance rates of *S. aureus* and CoNS. Data sources: CHINET Surveillance Reports of Bacterial Resistance 2009–2016.

The eight-year unbalanced panel dataset includes 100 records. Figure 1 exhibits the time series of average methicillin resistance rates of *S. aureus* and CoNS. The figure shows the much higher resistance rate of CoNS than that of *S. aureus* during the study period. Moreover, the resistance of *S. aureus* shows a decreasing tendency, whereas that of CoNS increases slightly with relatively larger fluctuation. Table 2 reports the summary statistics of the inputs. Of the labor inputs, the average number of nurses per 100,000 populations ranks the first, followed by pharmacists, infectious diseases physicians, and clinical microbiologists.

**Table 2:** Summary statistics of the input variables.

**Variables**	**Mean**	**Std. dev**.	**Min**	**Max**
*INF*	0.908	0.336	0.508	2.426
*PHR*	20.485	6.903	10.278	41.312
*MCB*	0.430	0.157	0.211	0.952
*NRS*	168.669	61.632	69.693	361.565
*BED*	363.814	88.514	181.708	546.711

*Std. dev. refers to standard deviation*.

**Figure 2:**
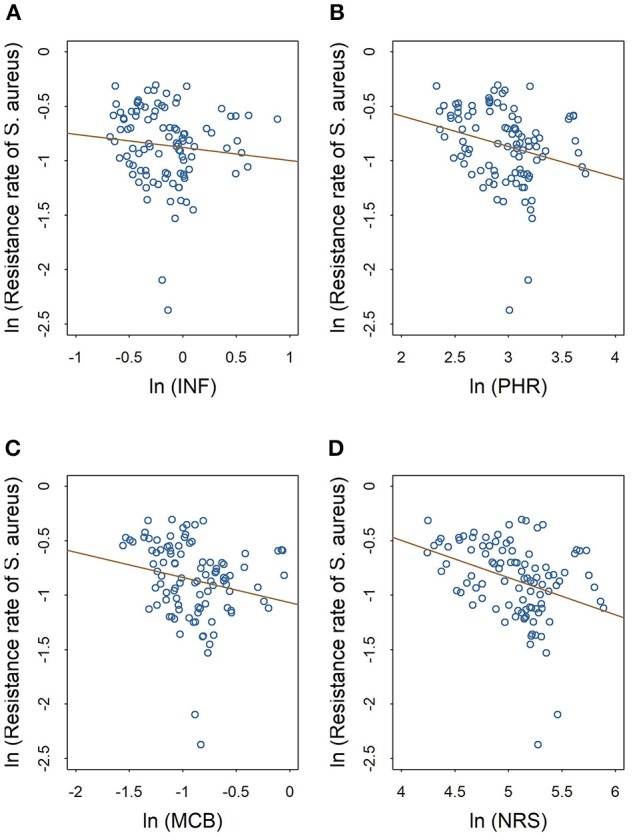
Linear relationships between medical staffing and methicillin resistance rates of S. *aureus*. The red line in each plot denotes the fitted line. **(A)** The linear relationship between the staffing of infectious diseases physicians and the methicillin resistance rate of *S. aureus*. **(B)** The linear relationship between the staffing of pharmacists and the methicillin resistance rate of *S. aureus*. **(C)** The linear relationship between the staffing of clinical microbiologists and the methicillin resistance rate of *S. aureus*. **(D)** The linear relationship between the staffing of nurses and the methicillin resistance rate of *S. aureus*.

**Figure 3:**
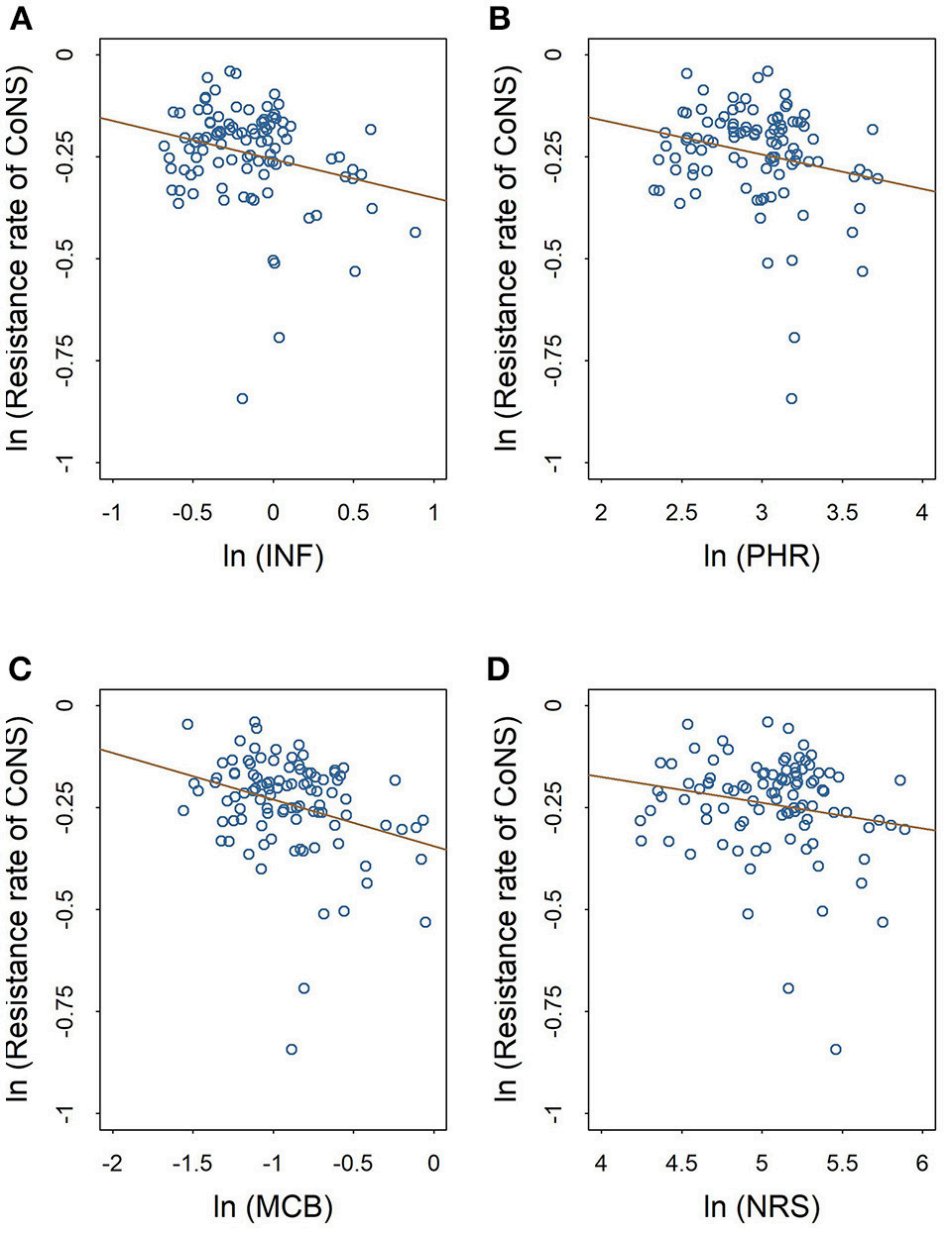
Linear relationships between medical staffing and methicillin resistance rates of CoNS. The red line in each plot denotes the fitted line. **(A)** The linear relationship between the staffing of infectious diseases physicians and the methicillin resistance rate of CoNS. **(B)** The linear relationship between the staffing of pharmacists and the methicillin resistance rate of CoNS. **(C)** The linear relationship between the staffing of clinical microbiologists and the methicillin resistance rate of CoNS. **(D)** The linear relationship between the staffing of nurses and the methicillin resistance rate of CoNS.

The scatter plots in Figures 2, 3 show the linear relationships between the numbers of different medical professionals (logarithmic form) and methicillin resistance rates of *S. aureus* and CoNS (logarithmic form), respectively. As the numbers of different medical professionals increase, the resistance rates of both pathogens decrease. Nevertheless, the relationships display greater variation in Figure 2 compared with those in Figure 3.

### Relationship between medical staffing and resistance of *S. aureus*

Table 3 displays the relationship between medical staffing and methicillin resistance of *S. aureus*. Coefficients for the dynamic models are presented in the last two columns [*m* = 1 in column (3) and *m* = 2 in column (4)] with the first two columns of coefficients indicating the results for the pooled ordinary least squares (OLS) and FE models. In the dynamic models, Sargan test accepts the validity of the instruments. However, Arellano–Bond test indicates a problem related to serial correlation in column (3) [*p* = 0.029 in *AR*(2)]. Hence, the dynamic model in column (3) is invalid and is thus, neglected.

**Table 3:** Relationship between medical staffing and methicillin resistance of *S. aureus*.

**Variable**	**Static**		**Dynamic**	
	**Pooled OLS (1)**	**FE(2)**	**GMM-SYS (3)**	**GMM-SYS (4)**
**LAGS OF DEPENDENT VARIABLE**				
ln*STW*_(*t*−1)_			0.307	0.287
			(0.254)	(0.270)
ln*STW*_(*t*−2)_				0.140
				(0.133)
**LABOR INPUT**				
ln*INF*	−0.036	−0.165	−0.184	−0.405
	(0.339)	(0.275)	(0.292)	(0.318)
ln*PHR*	−0.147	−0.602	−3.5*E*−4	0.106
	(0.398)	(0.630)	(0.510)	(0.465)
ln*MCB*	−0.282	−0.422	−0.463^*^	−0.561
	(0.367)	(0.318)	(0.259)	(0.350)
ln*NRS*	0.512	−0.350	0.394	0.648^**^
	(0.468)	(0.571)	(0.385)	(0.322)
**CAPITAL INPUT**				
ln*BED*	−0.371	1.256^**^	0.272	0.337
	(0.255)	(0.598)	(0.399)	(0.346)
**TECHNOLOGY**				
*T*	−0.084^*^	−0.074^*^	−0.088	−0.105^*^
	(0.046)	(0.041)	(0.054)	(0.059)
Constant	−0.677	−4.699	−4.158	−6.096^**^
	(3.47)	(3.084)	(3.237)	(2.947)
No. obs.	100	100	78	64
*F*	14.74^***^	5.70^***^		
Wald χ^2^			46.49^***^	104.86^***^
Arellano-Bond test				
AR(1) (*p*-Value)			0.039^**^	0.009^***^
AR(2) (*p*-Value)			0.029^**^	0.360
Sargan test (*p*-Value)			0.436	0.168

Significance:

*** *p < 0.01*,

** *p < 0.05*,

* *p < 0.10*.

*Robust standard errors in parentheses. Column (1) employs pooled ordinary least squares (OLS) model, while column (2) employs fixed–effect (FE) model. Column (3) and (4) employ dynamic models: Column (3) enrolls one lag of the dependent variable, while column (4) enrolls two lags. AR (1) and AR (2) examine the serial correlations of residuals in first and second differences, respectively*.

The pooled OLS and FE models suggest no statistically significant coefficients for medical staffing, whereas the dynamic model exhibits a significant but positive coefficient for the number of nurses (Coef. = 0.648; *p* = 0.044).

In addition to medical staffing, the technology exhibits significant and negative coefficients in the three models (Coef. = −0.084, −0.074, −0.105; *p* = 0.084, 0.081, 0.076, respectively). However, the number of beds shows a significant but positive estimate in column (2) (Coef. = 1.256; *p* = 0.048).

### Relationship between medical staffing and resistance of CoNS

Table 4 displays the relationship between medical staffing and methicillin resistance of CoNS. This table follows the same format as Table 3. In dynamic models, Arellano–Bond test indicates no problems relating to serial correlation. On the other hand, Sargan test accepts the validity of the instruments. Hence, both dynamic models are valid. More importantly, the significant dynamic effect is observed in column (4): the coefficient for the second lag of the resistance of CoNS (Coef. = −0.246; *p* = 0.005) suggests a substantial degree of persistence in resistance over time.

**Table 4:** Relationship between medical staffing and methicillin resistance of CoNS.

**Variable**	**Static**		**Dynamic**	
	**Pooled OLS (1)**	**FE(2)**	**GMM-SYS (3)**	**GMM-SYS (4)**
**LAGS OF DEPENDENT VARIABLE**				
ln*STW*_(*t*−1)_			0.055	0.009
			(0.086)	(0.133)
ln*STW*_(*t*−2)_				−0.246^***^
				(0.088)
**LABOR INPUT**				
ln*INF*	−0.042	0.050	−0.059	0.017
	(0.132)	(0.183)	(0.087)	(0.163)
ln*PHR*	0.178	0.231	0.075	0.184
	(0.158)	(0.260)	(0.244)	(0.165)
ln*MCB*	−0.169	−0.063	−0.191^*^	−0.351^***^
	(0.134)	(0.234)	(0.105)	(0.123)
ln*NRS*	−0.133	0.186	0.123	0.019
	(0.123)	(0.281)	(0.164)	(0.184)
**CAPITAL INPUT**				
ln*BED*	0.087	−0.362	−0.114	3.0*E*−4
	(0.092)	(0.227)	(0.219)	(0.181)
**MEDICAL TECHNOLOGY**				
*T*	0.007	0.016	0.008	0.001
	(0.013)	(0.018)	(0.011)	(0.010)
Constant	−0.800	0.122	−0.616	−1.251^*^
	(1.301)	(1.569)	(1.055)	(0.754)
No. obs.	100	100	78	64
*F*	6.56^***^	13.60^***^		
Wald χ^2^			45.04^***^	86.00^***^
Arellano-Bond test				
AR(1) (*p*-Value)			0.066^*^	0.011^**^
AR(2) (*p*-Value)			0.261	0.582
Sargan test (*p*-Value)			0.968	0.875

Significance:

*** *p < 0.01*,

** *p < 0.05*,

* *p < 0.10*.

*Robust standard errors in parentheses. Column (1) employs pooled ordinary least squares (OLS) model, while column (2) employs fixed-effect (FE) model. Column (3) and (4) employ dynamic models: Column (3) enrolls one lag of the dependent variable, while column (4) enrolls two lags. AR (1) and AR (2) examine the serial correlations of residuals in first and second differences, respectively*.

Similar to the results in Table 3, the pooled OLS and FE models suggest no significant coefficients for medical staffing. However, the number of clinical microbiologists exhibits significant and negative coefficients in both dynamic models (Coef. = −0.191, −0.351; *p* = 0.070, 0.004, respectively), indicating an association between the increased number of clinical microbiologists and decreased resistance of CoNS.

## Discussion

Using the eight-year provincial panel data, we investigate the relationship between medical staffing and antimicrobial stewardship performance in China. Several points are worthy of discussion.

The results indicate that the increasing number of clinical microbiologists is a significant predictor of better stewardship performance. This macro perspective finding is consistent with the results of prior micro studies. For example, O'Neill et al. (2005) conducted a study in the intensive care units of a 650-bed tertiary hospital in Ireland and discovered that recommendations by clinical microbiologists on antimicrobial treatment ensured a high degree of compliance with treatment modifications. Compared with other medical professionals, clinical microbiologists are more familiar with microbiological characteristics of pathogens. Their recommendations on antimicrobial treatment provide laboratory-based guidance on antimicrobial use, improving the physicians' prescribing habits and promoting the prudent use of antimicrobials (MacDougall and Polk, 2005; Morency-Potvin et al., 2017). Therefore, clinical microbiologists play an important role in ensuring antimicrobial stewardship performance.

The 10% increase in the number of nurses per 100,000 populations is significantly associated with 6.48% increase in the resistance of *S. aureus*. That is, a larger number of nurses is associated with poorer antimicrobial stewardship performance. This finding is contrary to our study hypothesis. We speculate on nurses' lack of antimicrobial knowledge that may underlie this finding. Although nurses are essential to antimicrobial stewardship, previous studies reveal that nurses receive limited education or training on antimicrobial stewardship, leading to the paucity of their related knowledge (Monsees et al., 2017). Besides, the fact that other medical professionals and even nurses themselves do not recognize nursing functions as tangible contributions to antimicrobial stewardship may also result in such finding (Olans et al., 2015; Monsees et al., 2017). Overlooking the role of nurses as antimicrobial stewardship allies may impede nurse participation in stewardship and collaboration with other medical professionals, accounting for negative effects on the stewardship performance.

Interestingly, in addition to clinical microbiologists and nurses, the numbers of infectious diseases physicians and pharmacists show no significant associations with antimicrobial stewardship performance. As mentioned in the introduction, the lack of abilities of both infectious diseases physicians and pharmacists poses a major concern in the antimicrobial stewardship in China (National Health and Family Planning Commission, 2016a; Zhang et al., 2018). This condition may explain the insignificant coefficients observed.

In addition to labor input, Table 3 shows that technological progress consistently features a significantly positive effect on stewardship performance. This finding highlights the importance of medical technology investment in antimicrobial stewardship, conforming to one of the strategic objectives in the global action plan on AMR by the World Health Organization (World Health Organization, 2015). However, the FE model in Table 3 indicates that a 10% increase in the number of hospital beds per 100,000 populations is significantly associated with 12.56% increase in the resistance of *S. aureus*. This finding may be attributable to the heavier workload of medical professionals. Liang et al. (2015) revealed that nursing units with heavier patient load upon nurses experienced higher patient mortality. Although a larger number of beds implies higher hospital capital, more patients must also be cared for. Hence, the heavier workload makes medical professionals spend more time on patient care rather than antimicrobial stewardship, thus, causing unsatisfactory stewardship performance. On the other hand, from a methodological perspective, such finding may be attributable to the model specification as the statistical significance is only found in the static model. This situation points out how different assumptions about the nature of data and the selection of an estimation technique can influence findings (Mark et al., 2004).

## Implications and limitations

This work reveals implications for health service researchers and health economists. Firstly, different from findings in previous studies, our study investigates the relationship between medical staffing and antimicrobial stewardship performance from a macro perspective. The present study goes beyond details of the behavior of individual units, such as hospitals, and thus increases understanding of the essence of the said relationship (Dornbusch et al., 2002). Hence, the study offers researchers a new perspective to investigate the roles of medical staffing in health care-related issues. Secondly, in addition to conventional static approach, the study also employs the dynamic approach which considers the dynamic nature of panel data and “feedback effects” to estimate the relationship between medical staffing and antimicrobial stewardship performance. This study is expected to serve as an empirical example of dynamic panel data modeling.

There are also implications for health policymakers on human resources allocation, utilization, and education. For instance, based on our findings, it is suggested that the government should ensure the adequate supply of clinical microbiologists. Education of laboratory medicine and medical microbiology should be promoted. The government should also provide other medical professionals, especially nurses, education or training on antimicrobial stewardship.

The present study has some limitations. Firstly, the relatively small data size may limit the robustness and statistical significance of the estimates. Secondly, we approximate the numbers of infectious diseases physicians and clinical microbiologists. Although approximation is a widely used approach in macro studies, it is not precise (Waldmann, 1992; Mark et al., 2004). Further studies should be conducted when more detailed data are obtained. Thirdly, although the total number of beds is the most commonly used proxy of hospital capital input in prior studies, it is somewhat imperfect as it focuses more on the inpatient aspect than the outpatient one (Suraratdecha and Okunade, 2006). Qualitative studies should be conducted to seek a more appropriate indicator of hospital capital input. Fourthly, the present study focuses on the effectiveness of medical staffing while paying little attention to the costs. Increasing medical staffing may improve antimicrobial stewardship performance but also implies that hospitals must pay more salaries. Hence, if more data are available, the costs of antimicrobial stewardship should be studied to provide further evidence and guidance on medical staffing allocation, utilization, and management.

## Conclusions

The present study analyzes the relationship between medical staffing and antimicrobial stewardship performance in China from a macro perspective. Both static and dynamic panel data approaches are employed. The results indicate that increasing the number of clinical microbiologists is a significant predictor of better stewardship performance, empirically demonstrating the important role of these professionals. However, a larger number of nurses is significantly associated with poorer performance. This result may be attributed to the nurses' lack of related knowledge and their unrecognized functions in antimicrobial stewardship. Based on these findings, policy implications are given, including ensuring the availability of clinical microbiologists in adequate numbers and providing other medical professionals, especially nurses, education or training on antimicrobial stewardship.

## Data availability statement

The datasets generated during and/or analyzed during the current study are available in *China's Statistics Yearbooks of Health and Family Planning* 2010–2017, and *CHINET Surveillance Reports of Bacterial Resistance* 2009–2016.

## Author contributions

JL conducted the study design, statistical analysis, data interpretation, and drafted the manuscript. CY and CL made significant contributions to the data interpretation and manuscript writing. CY also provided essential assistance during the revision process. YT and XZ provided assistance in study design and manuscript writing. All authors reviewed the manuscript.

### Conflict of interest statement

The authors declare that the research was conducted in the absence of any commercial or financial relationships that could be construed as a potential conflict of interest.
